# The NF-κB signaling network in the life of T cells

**DOI:** 10.3389/fimmu.2025.1559494

**Published:** 2025-04-30

**Authors:** Mark A. Daniels, Emma Teixeiro

**Affiliations:** ^1^ Department of Molecular Microbiology and Immunology, School of Medicine, University of Missouri, Columbia, MO, United States; ^2^ Roy Blunt NextGen Precision Health Building, University of Missouri, Columbia, MO, United States

**Keywords:** NF-kB, T cell, signaling, differentiation, network

## Abstract

NF-κB is a crucial transcription factor in lymphocyte signaling. It is activated by environmental cues that drive lymphocyte differentiation to combat infections and cancer. As a key player in inflammation, NF-κB also significantly impacts autoimmunity and transplant rejection, making it an important therapeutic target. While the signaling molecules regulating this pathway are well-studied, the effect of changes in NF-κB signaling levels on T lymphocyte differentiation, fate, and function is not fully understood. Advances in computational biology and new NF-κB-inducible animal models are beginning to clarify these questions. In this review, we highlight recent findings related to T cells, focusing on how environmental cues affecting NF-κB signaling levels determine T cell fate and function.

## Introduction

Since its discovery in 1986 (1), the transcription factor NF-κB has been associated with numerous cellular processes, including cell function, differentiation, and stemness (2–5). NF-κB plays a crucial role in opposing cell responses like survival and cell death and influences specific T cell differentiation paths. While subunit composition of NF-κB heterodimers can sometimes explain these outcomes, the regulation of this transcription factor remains complex, involving transcriptional and epigenetic levels. NF-κB is induced via a signaling cascade converging at the IKK complex, composed of catalytic subunits IKKα and IKKβ, and the regulatory subunit NEMO (IKKγ) (6). The upstream signals activating the IKK complex vary with environmental cues (3, 7, 8). Additionally, NF-κB alone is often insufficient for gene regulation; it relies on interactions with other transcription factors, its affinity for binding sites at gene promoters or enhancers, and its influence on chromatin remodeling (9). Modeling studies indicate that the timing and levels of NF-κB signaling are critical in determining T cell responses and fate (10–13). This review examines how environmental cues utilize NF-κB signaling to regulate T cell lymphocytes, highlights unresolved questions, and explores implications for therapeutic interventions.

## The NF-κB signaling pathway

**Figure 1:**
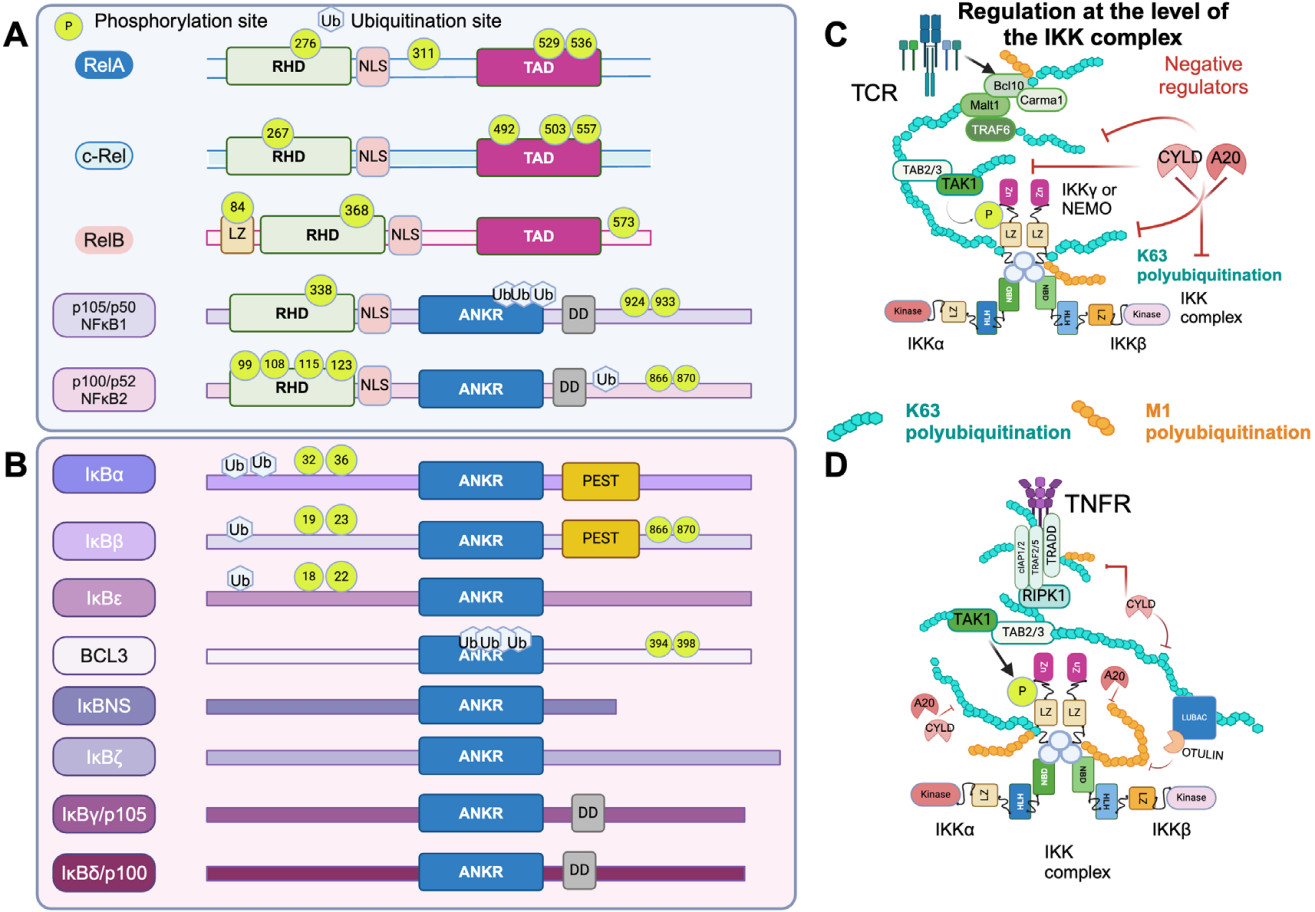
The NF-κB signaling network. **(A)** NF-κB subunits or Rel Homology domain proteins of the NF-κB family. RHD or Rel homology domain determines dimerization with other members of the NF-κB/Rel protein family; NLS or nuclear localization sequence, which also comprises the site for inhibitory interactions with IkB. TAD, or transactivation domain. LZ or leuzin zipper. AnkR or ankyrin repeats domain. DD or death domain. Regulatory phosphorylation sites are denoted by their position in green. Ubiquitination sites (ub) are also shown. **(B)** Inhibitors of the NF-κB family. The ankyrin repeats in these proteins mediate the binding to NF-κB dimers. The N terminal part of the IκB proteins contain two Ser residues that when phosphorylated allow for quick ubiquitination and degradation of the protein. This is true for the typical IκB proteins but not for the atypical (IκB_NS_, IκBζ, IkBη and BCL3). The C-terminal regions of p105 and p100 aside from the ankyrin repeats, they also have a death domain. These regions function as IκB for their RHD domain by forming large complexes with Rel proteins. The death domain can undergo cotranslational processing by the 26S proteosome to produce p50 or p52. DD domains also can allow binding to other proteins with complimentary DD domains enabling the assembly of signaling complexes involved in cell death pathways. Some IκB members also have a PEST domain rich in Proline, glutamic acid, serine and threonine. The PEST domain acts as a signal for rapid degradation. **(C)** The cartoon illustrates the regulation of the IKK complex by the positive actions of signaling intermediates downstream of the T Cell Receptor (TCR) and the TNF receptor. When the TCR binds to antigen presented in the context of MHC, it leads to the phosphorylation of tyrosines in the CD3 chains of the TCR/CD3 complex, facilitating the recruitment of the kinase ZAP-70. ZAP-70 then phosphorylates its substrates, LAT and SLP76, forming the LAT/SLP76 signalosome. Once phosphorylated, this complex enables the recruitment of Vav, which mediates cytoskeleton reorganization and translocation of PKCθ to the membrane. At the membrane, PKCθ phosphorylates CARMA1, leading to the formation of the CBM complex (CARMA1, BCL10, and MALT1). This complex then oligomerizes and recruits the ligase TRAF6 and the kinase TAK1. A dimer of IKKγ (NEMO) binds to IKKα and IKKβ to form the IKK complex. The linear and K63 polyubiquitination of NEMO allows for the activation of the IKK complex, further supported by phosphorylation by TAK1. IKK activation leads to the phosphorylation of the NF-κB subunit p65 and the inhibitory protein IκBα. K63, K11, and M1 ubiquitination represent key processes in NF-κB signaling that enable the formation of non-degradative ubiquitin chains, which are critical for the recruitment of various components of the NF-κB signaling cascade. **(D)** Downstream of the TNF receptor, TRAF complexes recruit RIPK1. The LUBAC complex catalyzes the M1 polyubiquitination of NEMO which allows for activation of the IKKc. Negative regulation at the level of the IKKc signalosome can occur through various mechanisms, including dephosphorylation and deubiquitination by phosphatases such as PP2A and PP2C, or by deubiquitinases like A20 and CYLD (K63 polyubiquitin chains), and Otulin (M1 polyubiquitin chains). This figure has been created with BioRender.com.

**Figure 2:**
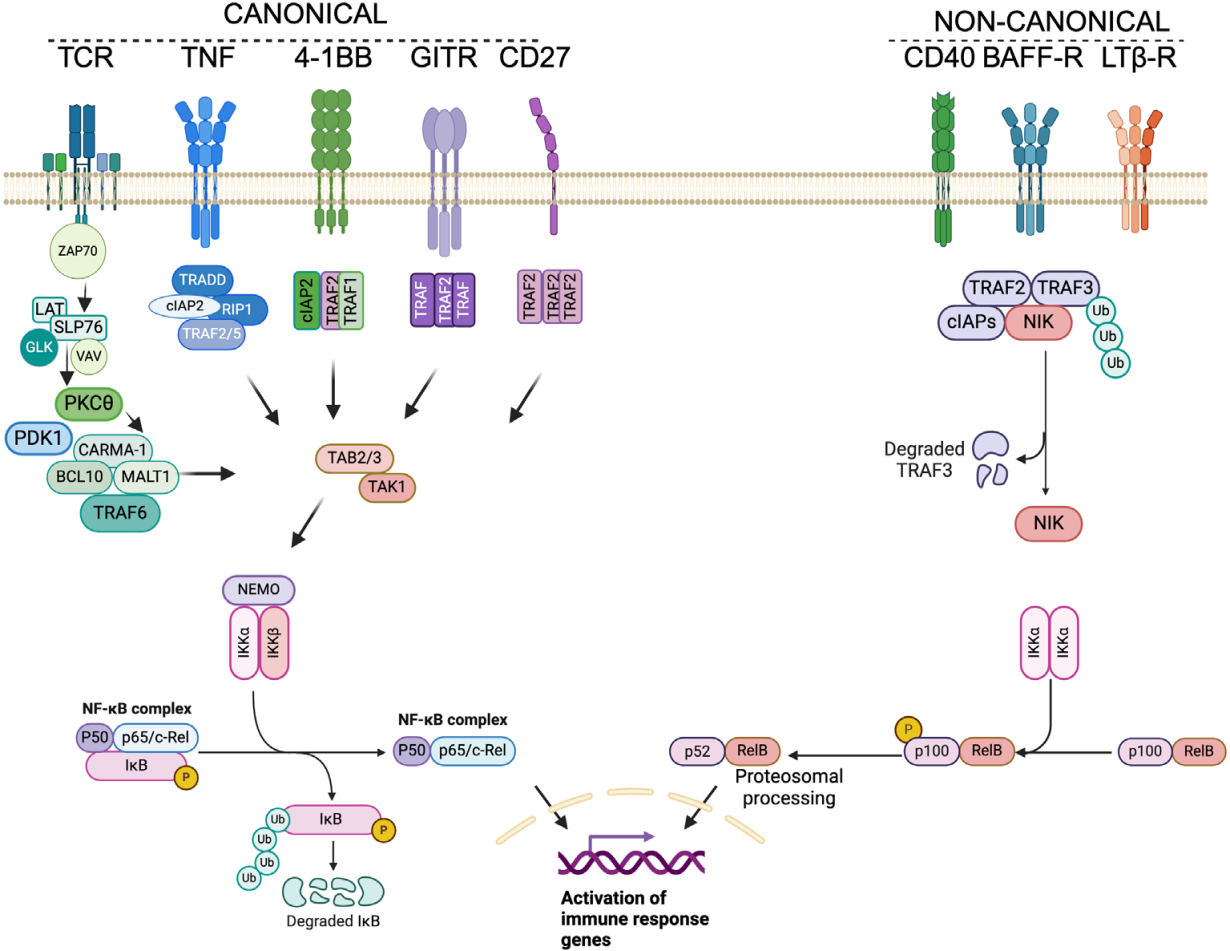
Canonical and non-canonical NF-κB signaling in T cells. Left. This cartoon illustrates the membrane receptors involved in delivering canonical NF-κB signaling in T cells, including the T Cell Receptor (TCR) and members of the TNF receptor superfamily. TCR stimulation leads to the recruitment of the kinase ZAP70 to the phosphorylated ITAMs of the CD3 chains within the TCR/CD3 complex. This recruitment enables ZAP70 to phosphorylate LAT and SLP76, forming the LAT/SLP76 signalosome, which subsequently recruits the guanine exchange factor Vav and the kinase GLK. These components contribute to the membrane translocation and activation of PKCθ. Next, PKCθ phosphorylates CARMA1, resulting in the formation of the CBM complex (comprised of CARMA1, BCL10, and MALT1). The subsequent recruitment of the ligase TRAF6 and the kinase TAK1 into the NF-κB signalosome leads to the ubiquitination of NEMO and the phosphorylation of IKKα and IKKβ. The active IKK complex (NEMO-IKKα-IKKβ) phosphorylates IκBα, which retains NF-κB heterodimers (p50-RelA or p50-c-Rel) in the cytosol, targeting IκBα for degradation. This process allows free NF-κB dimers to translocate to the nucleus and drive gene expression. TNF receptor signaling converges with TCR signaling at the level of TAK1 and the IKK complex; however, each receptor leads to the assembly of specific signalosomes where TRAF proteins play critical roles. Right. Non-canonical NF-κB signaling differs from canonical signaling in terms of the NF-κB dimers (p52-RelB), the IKK complex (which consists of a dimer of IKKα), the kinase activity (NIK), and the membrane receptors depicted in this cartoon. Additionally, while not included in the figure, evidence exists for crosstalk between both pathways. This figure was created using BioRender.com.

NF-κB is a family of transcription factors comprised of several members: RELA (p65/RelA), c-REL (c-Rel), RELB (RelB), NFKB1 (p105/p50), and NFKB2 (p100/p52) (**Figure 1A**). These members form homo- or heterodimers via an N-terminal Rel homology domain (RHD) (14). Canonical and non-canonical signaling pathways enable different membrane receptors to induce NF-κB nuclear translocation and regulate its transcriptional activity (**Figure 2**). The canonical NF-κB pathway induces the formation of p50-RelA and p50-c-Rel dimers (15, 16), which in resting cells are sequestered in the cytoplasm by IκB proteins (IκBα, IκBβ, IκBγ, IκBϵ) (17–23) or regulated in the nuclei by the atypical IκB proteins IκB_NS_ (24, 25), IκBζ (26) IkBη (27) and BCL3 (28, 29). Meanwhile, exposure to environmental stimuli induces the activation of the multi-subunit IκB kinase (IKK) complex which drives the phosphorylation, polyubiquitination and degradation of IκB proteins in the 26S proteasome (6, 30). This allows NF-κB heterodimers to translocate to the nucleus to regulate gene expression. In contrast to this, the non-canonical pathway induces p52-RelB heterodimers through the activation of NF-κB-inducing kinase (NIK), which phosphorylates IKKα dimers, leading to p100 processing into p52 (31–33). This pathway is triggered by stimuli such as LIGHT, TWEAK, BAFF, RANKL, and CD40L, while canonical signaling is activated by antigens, CD28, 4-1BB, GITR co-stimulators, TNF, and IL-1 (3, 8, 34), (**Figures 1**, **2**). Notably, while all these receptors share the same downstream signaling steps following IKK complex/IKKc activation (canonical or non-canonical), they differ upstream (**Figure 2**). Modulation of NF-κB signaling levels lies both in the diversity of intermediates driving IKKc activation and the regulated expression of antigens, TNFRs, and cytokine receptors. It is also important to consider the distinct and overlapping roles of canonical and non-canonical NF-κB signaling. Activation of the canonical pathway has been classically associated with inflammation, while activation of the non-canonical pathway regulates cell development and organogenesis. Both pathways have been considered to signal independently (35). However, extensive overlap exists at the level of p50-RelA, IκBδ and p100 (36, 37). Similarly, canonical control of p52-RelB can occur via the p50-RelA dependent expression of p100 and RelB (36, 37). At the level of NF-κB subunit dimerization, RelA can bind to RelB and prevent its DNA binding (38). Upstream, crosstalk has also been reported at the level of RIPK1 (39, 40) and NIK (41, 42).

There is still a significant gap in understanding how T cells integrate canonical and non-canonical signals from different receptors at the membrane level. Additionally, the ways T cells respond to varying NF-κB signaling levels according to tissue context and how these variations influence T cell outcomes remain unclear. In the following sections, we will discuss how NF-κB signaling regulates T cell development and its resulting effects.

## Mechanisms by which NF-κB signaling regulates thymocyte development

In the thymus, immature T cells, or thymocytes, commit to the T cell lineage and develop tolerance to self-antigens during thymic development. T cell progenitors differentiate into CD4 and CD8 double negative thymocytes (43). They rearrange the T cell receptor (TCR) beta chain and express a pre-TCR. During this phase, NF-κB signaling regulates pre-TCR signaling, providing survival signals for thymocytes with productive TCR beta chain rearrangements (44). This is followed by the rearrangement of the TCR alpha chain and the expression of CD4 and CD8 coreceptors, resulting in the development of double-positive (DP) thymocytes (43).

DP thymocytes, in turn, recognize self-antigens and MHC molecules (class I or class II) on the surface of cortical thymic epithelial cells via their TCRs and undergo thymic selection. The affinity of this interaction determines thymocyte fate. DP thymocytes whose TCRs bind to pMHC with insufficient affinity or excessively high affinity undergo death by neglect or are negative selected respectively. Only those with intermediate affinity to self-peptide-MHC (pMHC) interactions are positively selected and further differentiate into single-positive (SP) CD4^+^ or CD8^+^ thymocytes (43). The role of NF-κB signaling in thymic development has been investigated using transgenic mice expressing either the super-repressor form of IκBα (*Nfkbia*-SR) or the constitutively active form of IKKβ (*Ikbkb*-CA) (45). Data from these studies indicate that increased NF-κB signaling in double-positive (DP) thymocytes recognizing MHC class I, skews them towards the negative selecting threshold, resulting in their death. Conversely, decreased NF-κB signaling prevents DP thymocytes from reaching the positive selecting threshold, leading them to succumb to death by neglect. Interestingly, these studies also reported that DP thymocytes recognizing MHC class II, do not signal through NF-κB and thus, inhibition of NF-κB signaling in these cells do not affect thymic selection (45). Altogether, these studies demonstrate that NF-κB signaling is crucial for establishing the signaling thresholds that determine positive and negative selection, particularly for the selection of CD8 SP thymocytes (45).

Other studies that used transgenic mice expressing mutant IkBα or a conditional deletion of IKKβ or NEMO/IKKγ have also showed that reduced NF-κB signaling affects CD8 SP T cells beyond thymic selection (46–48). Specifically, inhibition of NF-κB leads to a significant decrease in CD8 SP T cells in the thymus. However, it remains unclear whether this defect is due to induction of cell death or a defect in cell survival. Supporting the latter, active NF-kB levels are much higher in CD8 than in CD4 SP thymocytes, while the pro-survival factor Bcl2 is expressed at lower levels in CD8 SP cells (45). These data suggest that CD8 SP survival may require different NF-kB signaling levels than CD4 SP cells, making CD8 SP cells more susceptible to the effects of NF-κB inhibition.

NF-kB signaling intermediates upstream of the IKK complex are also important in thymic development. The kinase TAK1 (**Figure 2**) specifically regulates the maturation and survival of single-positive (SP) thymocytes, rather than their selection (49). Hogquist’s group demonstrated that TNF-dependent TAK1→ NF-kB signaling is essential for cell survival at the SP stage and late thymic maturation (50). Their findings indicate that TCR-dependent signaling is not involved in this process. This agrees with other studies indicating that TCR-proximal NF-kB intermediates (CARMA1, BCL10, and MALT1) do not affect thymic development (51–53). Notably, TAK1 can also function independently of NF-kB by mediating Type I IFN signals, enhancing thymocyte responsiveness to inflammatory cytokines (50). Additionally, recent work by Seddon’s group has shown that IKK-mediated repression of RIPK1 is a crucial link between TNF receptor signaling and TAK1. This link is necessary to support post-selection SP survival and maturation (54). Thus, environmental factors beyond just antigens tightly control CD8 SP survival/late maturation through mechanisms involving specific NF-kB signaling intermediates, making T cells more responsive to inflammatory signals.

Noteworthy, downstream of the IKK complex in the NF-kB signaling cascade, recent studies using CD4cre-inducible models of RelA and c-Rel deletion have shown that these NF-kB subunits are not necessary for thymic development (55). Hence, while the role of the NF-κB signaling cascade in thymic selection and SP maturation is evident, data suggests that it does not follow conventional transcriptional NF-κB mechanisms. Moreover, depending on the stage of development (thymic selection versus SP maturation) unique NF-κB signaling intermediates play predominant roles.

## NF-κB signaling and T cell homeostasis

Single positive T cells migrate to the periphery, where they can persist for months as naive T cells. The homeostasis of these cells relies on their survival and low turnover properties (56). The role of NF-κB signaling in the survival and homeostasis of peripheral T cells has been elucidated through mouse models expressing T cell-restricted conditional transgenes. These models include those with inactive kinase forms (achieved by replacing the serines in the kinase activation loop with alanines to eliminate inducible IKK activity) (57), and those expressing constitutively active forms of IKKβ (where the activation loop serine residues (Ser 177 and 181) are substituted with glutamic acid) (58). Additionally, some models have employed degradation-resistant or truncated forms of IkBα (59–61). Inhibition of canonical NF-κB signaling in any of these models leads to significant reductions in peripheral T cell populations, including both naive and memory T cells (48). However, it has remained unclear whether the extent of this decrease is influenced by defects in thymic selection, inefficient transgene expression, or specific targeting of IKKα, IKKβ, or IkBα (62). Recent investigations have revisited the role of IKKβ, and NF-κB signaling in T cell homeostasis using tamoxifen-inducible and temporally controlled deletion of IKKβ (63). These studies indicated that IKKβ expression is essential for the upregulation of IL-7R on selected T cells as they exit the thymus (63). This report showed that the induced expression of IL-7R via TNF and CD70/CD27-dependent NF-κB signaling is transient but, at the same time, crucial for recent thymic emigrants to fully mature into functional peripheral T cells (63). Interestingly, in contrast to previous reports, these studies found that IKKβ expression did not regulate IL-7R expression or the survival of peripheral naive T cells in immunocompetent mice (63). This suggests that IKKβ regulates recent thymic emigrants transition into naive T cells in an antigen receptor-independent manner, consistent with data from mice deficient in proximal components of TCR-dependent NF-κB induction (48, 64).

**Figure 3:**
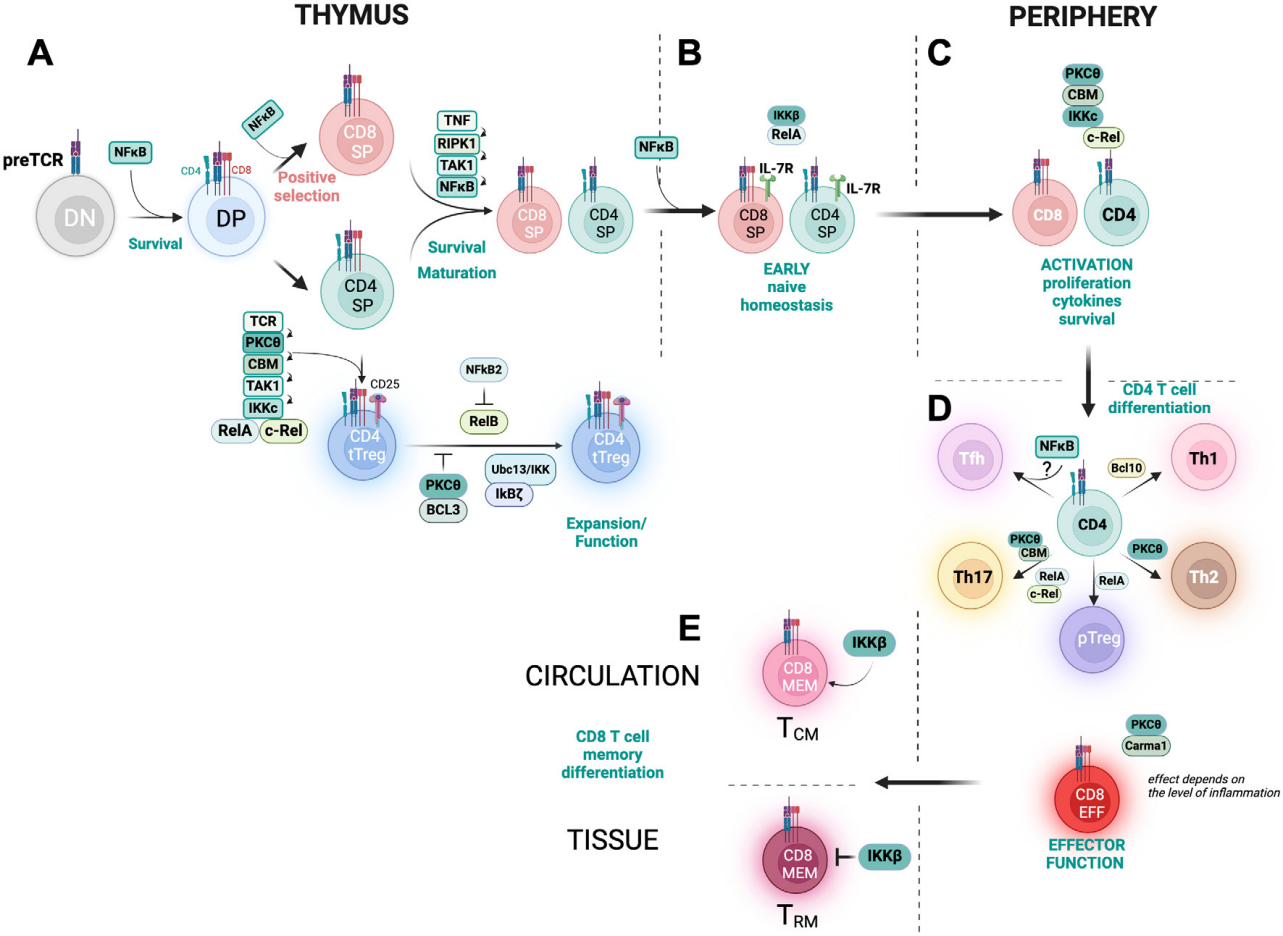
Roles of NF-κB signaling intermediates in T cell differentiation. This cartoon shows the phases of CD4 and CD8 T cell differentiation in the thymus **(A, B)** and the periphery **(C-E)** highlighting the roles of different components of the NF-κB signaling network. DN, double negative thymocyte. DP, double positive thymocyte. SP, single positive thymocyte. tTreg refers to thymic T reg while pTreg refers to peripheral Treg. Tfh is follicular helper CD4 T cell, Th is a CD4 T helper cell. CD8EFF is an effector CD8 T cell while CD8MEM is a memory CD8 T cell. TCM is central memory T cell. TRM is tissue resident memory T cell. This figure has been created with BioRender.com.

Further studies have explored the roles of NF-κB subunits in the survival of mature T cells. Research using p50-c-Rel double-deficient mice found no defects in thymic development, T cell maturation, or naive T cell homeostasis (65). This prompted further investigations into the outcomes observed in c-Rel, p65/RelA and RelB knockout mouse models, where multiorgan inflammation occurs, making it unclear whether defects arise from the stroma or the T cells themselves (66–68). More recent studies using CD4 cre-inducible models for RelA and c-Rel deletion have supported earlier findings seen with IKKβ and IkBα transgenic models (48, 59). These studies revealed a significant reduction in the populations of peripheral naive CD4 and CD8 T cells in the absence of RelA, while c-Rel deficiency did not have the same effect (69). Furthermore, RelA deficiency was linked to lower levels of Ki67-positive proliferating cells and reduced production of key cytokines, such as IFN-γ and IL-2, upon PMA/Ionomycin stimulation (69). This regulation often works in conjunction with AP-1 family members, like Jun and BATF, affecting crucial T cell proteins such as CD83, ICOS, and 4-1BB (69). Overall, these findings highlight the critical role of RelA in T cell homeostasis, as it regulates many genes essential for T cell activation through transcriptional control (**Figure 3B**) (69).

## NF-κB signaling and mature T cell responses: cell division, apoptosis and effector function

Naive CD4 and CD8 T cells recognize cognate antigens and receive costimulatory signals from dendritic cells (DCs) in draining lymph nodes, leading to their activation (70). This activation triggers the early upregulation of CD69, a crucial factor for retaining naive T cells in the lymph node to facilitate continued activation (70). Subsequently, T cells increase the expression of the high-affinity IL-2 receptor alpha chain (CD25) and the pro-survival cytokine IL-2 (70). These processes occur within the first 24 hours post-activation, supporting T cell division and survival, and are influenced by NF-κB signaling (70, 71). Indeed, naive T cells deficient in the NF-κB subunits RelA and c-Rel exhibit impaired proliferation and cytokine production after stimulation (72, 73), (**Figure 2**). Additionally, defects in upstream signaling components, such as IKKα, IKKβ, and CARMA1 lead to inadequate T cell proliferation (74, 75) and increased apoptosis upon activation (76, 77). The deletion of other members of the CBM complex, including BCL10 and MALT1, as well as PKCθ, similarly hampers T cell proliferation, particularly in CD4 T cells, These CD4 T cell proliferation defects cannot be rescued by exogenous IL-2 (although it enhances their survival). By contrast, in CD8 T cells, deficiency of these NF-κB early signaling intermediates raises the threshold of TCR signaling required for proliferation, resulting in impaired CD8 T cell proliferation even with strong agonistic TCR stimulation (78).

Regarding apoptosis, BCL10 does not have a significant role, whereas PKCθ and CARMA1 are more prominently involved (**Figure 2**). Specifically, PKCθ is essential for the expression of Fas ligand (FasL), which mediates activation-induced cell death (AICD) (79). Conversely, the role of MALT1 is more complex. It interacts with Caspase-8 during TCR engagement, limiting its autoproteolytic processing and apoptotic activity without affecting c-Flip processing. This suggests that MALT1 contributes to both T cell proliferation and survival through NF-κB signaling (80).

Costimulation, particularly via CD28, is crucial for T cell-dependent NF-κB activation, which occurs through the induced membrane recruitment and enzymatic activity of PKCθ and PDK1 (81) (**Figure 2**). Depletion of PDK1 disrupts the synergy between TCR and CD28 signals, leading to impaired NF-κB induction. Specifically, the absence of PDK1 hinders PKCθ activation and prevents the formation of the CBM complex (81). However, other functions of PKCθ, such as activating the p38 MAPK, JNK, and Calcium/NFAT pathways, remain unaffected (81). Consequently, while certain aspects of T cell activation—including proliferation, CD25 expression, and IL-2 production—rely on PDK1, neither apoptosis nor survival of CD4 or CD8 T cells is significantly compromised in its absence (81).

Additionally, the MAPK GLK is recruited to the TCR/LAT/SLP76 signalosome, mediating the phosphorylation and activation of PKCθ (S5388), which is necessary for NF-κB induction (82), (**Figure 2**). Although GLK-deficient T cells demonstrate impaired proliferation and cytokine production, their involvement in survival or apoptosis has not been assessed (82). Furthermore, while GLK does not alter ERK or Calcium/NFAT signaling, the specific role of GLK in JNK and p38 MAPK signaling remains unexamined. Thus, similar to PDK1, GLK may not be critical for T cell survival if these pathways remain active (82) (**Figure 2**).

Altogether, this body of work supports the notion that TCR and CD28-dependent NF-κB signaling intermediates play a vital role in regulating early T cell activation and responses related to proliferation and cytokine-driven survival (**Figure 3C**).

## NF-κB signaling, T cell effector differentiation and function

### CD4 T cell differentiation

As T cells proliferate, they undergo a differentiation process that transforms them into specialized effector T cells and long-lived memory T cells. This differentiation is crucial for establishing long-term protective immunity against pathogens and tumors (83). Naive CD4 T cells initially differentiate into effector precursors in response to antigen recognition, costimulation, and specific cytokine signals. These three inputs drive the expression of unique transcription factors that act as master regulators of T cell fate decisions and guide helper T cell (Th) subset lineage commitment (84). CD4 T cell differentiation is intricately adapted to the type of pathogen and the innate signals it triggers. For example, intracellular pathogens initiate Type I immune responses characterized by the generation of Th1 CD4 effector T cells, which secrete hallmark cytokines IFN-γ, IL-2, and TNF. Naive T cells differentiate into Th1 effectors following initial stimulation through their TCR, CD28, and IL-12 signals (84–89). While TCR and CD28 signaling activate the transcription factors NF-κB, NFAT, and AP-1, IL-12 signaling activates STAT4 (84–89). These transcription factors cooperate to upregulate the expression of T-bet, the master transcription factor of the Th1 subset, which is critical for the expression of IFN-γ and other transcription factors, including Hlx and Runx3 (84–89). Strong TCR signaling also promotes the expression of Blimp-1, IRF4, and the IL-2-dependent transcription factor STAT5, facilitating the re-expression of IL-12R and inducing the chemokine receptor CXCR3. This results in the migration of primed Th1 cells to tissues, where they further differentiate into committed Th1 effectors (84–89). Concurrently, transcriptional networks that promote alternative Th cell fates (e.g., GATA3, RORγt, or Bcl6) are repressed (84–89). In contrast to this, weak TCR signaling drives the expression of the master T follicular helper (Tfh) regulator Bcl6, results in low levels of IL-2R/STAT5 signaling and supports the expression of CXCR5 (84–89). This directs primed CD4 T cells toward the T-B border of the lymph node, where they interact with B cells and specific DCs to receive ICOS/ICOSL signals (84–89). In conjunction with IL-6, IL-21, and IFNγ signals, these interactions enhance Bcl6 levels while inhibiting the expression of master transcription factors associated with other Th subsets (84–89). Ultimately, this results in the generation of differentiated PD-1^hi^ germinal center Tfh cells that provide critical assistance to B cells for isotype switching and affinity maturation (84–89). In contrast to Type I immune responses, Type II responses are elicited in response to extracellular parasites and allergens, driving the generation of Th2 cells through T cell receptor (TCR) engagement, costimulatory signals, and exposure to IL-4 (84–89). IL-4 is essential for the expression of GATA3, the master transcription factor that plays a critical role in the secretion of Th2 signature cytokines, including IL-4, IL-5, and IL-13. Extracellular bacteria and fungi, however, elicit Type III immune responses characterized by the differentiation of Th17 cells (84–89). This process is mediated by antigen recognition, costimulatory signals, and exposure to cytokines such as TGF-β, IL-6, IL-21, and IL-1β. These signals drive the expression of the Th17 master transcription factor RORγt and STAT3, regulating the production of IL-17 and IL-22 (84–89).

Numerous studies conducted in the late 1990s and early 2000s established that both canonical and non-canonical NF-κB signaling pathways were crucial for Th1 and Th2 differentiation, albeit acting at different stages. T cells deficient in IκBα degradation, as well as those lacking c-Rel or RelA expression, display impaired Th1 responses, independent of T-bet expression. Instead, both NF-κB subunits directly regulate IFNγ expression, potentially in cooperation with STAT4. Notably, p50 deficiency did not influence T-bet or IFN-γ expression in CD4 T cells, suggesting that RelA may regulate IFNγ through homo- or heterodimers with other NF-κB subunits (90). RelB, however, is required for T-bet expression, likely through STAT4 induction (91). Other studies have shown that NF-κB signaling was also instrumental in Th2 differentiation (92). Specifically, p50/Rel is necessary for GATA3 induction mediated by IL-4, while RelA/p65 and c-Rel worked in conjunction with NFAT to support IL-4 induction in a TCR-dependent manner (90, 92–95). The roles of NF-κB subunits in Th17 differentiation have been corroborated by various studies, demonstrating the importance of both RelA and c-Rel in IL-17 expression, as well as for c-Rel’s transcriptional activity in promoting RORγt expression (96). Additionally, the atypical IκB protein IκBζ (97) (**Figure 1B**) is involved in Th17 differentiation. It cooperates with RORγt and RORα to drive IL17A expression, and its deficiency causes a defect in Th17 differentiation and a resistance to EAE (97).

Together these foundational studies established a link between NF-κB and CD4 T cell differentiation. However, they also had technical limitations, such as reliance on germline or conditional gene deletion and *in vitro* conditions. These factors made it difficult to gain a clear understanding of the timing and specific contributions of each subunit during the differentiation of T helper subsets. To address this, the roles of c-Rel and RelA in CD4 T cell differentiation have recently been revisited using novel tamoxifen-inducible mouse models that specifically delete c-Rel or RelA expression in naive CD4 T cells (69). These studies have demonstrated that c-Rel is more critical than RelA in regulating CD4 T cell proliferation (particularly under conditions of supra-optimal TCR and costimulatory signaling in the absence of IL-2) as well as in the expression of IL-2 and TNF in both human and murine cells (69). Additionally, unlike findings from earlier studies, this recent report shows that neither c-Rel nor RelA are essential for the expression of T-bet, GATA3, IFNγ, or IL-13, and thereby not critical for Th1 and Th2 cell differentiation. By contrast, both NF-κB subunits are essential for the expression of the Th17 master transcription factor RORγt, with RelA also playing a role in regulating IL-17 expression (**Figure 3D**). Surprisingly, CD4-restricted ablation of RelA (but not c-Rel) provides protection against experimental autoimmune encephalomyelitis (EAE),a model of autoimmune disease that heavily relies on the presence and function of pathogenic Th17 cells (69).

Although these elegant NF-κB -inducible models have not yet been employed to study T follicular helper (Tfh) cell differentiation, recent reports suggest a potential role for NF-κB signaling in this process. For instance, patients with a truncated mutation of NFKB2 exhibit a reduced presence of Tfh and T follicular regulatory T (Tfr) cells, along with impaired germinal center (GC) potential. It remains unclear whether this loss is independent of associated B cell defects caused by the truncated mutation (98). By contrast, non-canonical NF-κB signaling plays an important role in the expression of proteins that facilitate Tfh differentiation, such as ICOS ligand (ICOSL) in BAFF-stimulated B cells (99). Similarly, CD4 T cells deficient in p50 demonstrate defects in CXCR5 expression, which is critical for Tfh cell localization within the germinal center (100). Despite these findings, it is still unknown whether other canonical NF-κB subunits are essential for Tfh development, or whether NF-κB plays a role earlier in Tfh priming and/or later during the differentiation process in a T cell-intrinsic manner. Moreover, significant knowledge gaps persist regarding the impact of T cell-intrinsic NF-κB signaling on affinity maturation and B cell fate or function. Addressing these questions will enhance our understanding of Tfh biology and its implications for immune responses.

While the referenced studies have established a direct correlation between NF-κB epigenetic and transcriptional activity and CD4 T cell effector function and differentiation, the role of upstream intermediates in the NF-κB signaling cascade should not be overlooked. Caution is warranted, however, as upstream components of the NF-κB cascade often engage in significant crosstalk with other signaling pathways (**Figure 2**). Investigations using CARMA1-deficient T cells have confirmed that TCR-proximal NF-κB signaling is crucial for the Th17 phenotype and function (IL-17, IL-22, IL-21, IL23R, CCR6 expression) but not for Th1 or Th2 differentiation. Interestingly, mice with CARMA1-deficient CD4 T cells exhibit resistance to experimental autoimmune encephalomyelitis (EAE), yet their T cells do not show impaired expression of RORγt, Ahr, IRF4, or STAT3/STAT5 signaling (77). This finding suggests that CARMA1-dependent NF-κB signaling is required following the initial priming and polarization of Th17 cells and is essential for completing Th17 differentiation.

Other studies have also shown that a deficiency in the formation of the CBM complex lead to defects in the induction of Th1 and Th17 subsets. Mechanistically, this did not seem related to IKK and NF-κB but rather to the ability of the CBM complex to facilitate efficient glutamine uptake through ASCT2 and activation of mTORC1 upon TCR/CD28 stimulation (101). On the other hand, T cells expressing constitutively active MALT1 show impaired NF-κB signaling but exhibit continuous degradation of the downstream target Roquin-1, leading to unregulated differentiation of naive T cells into Th1 and Th17 effector cells. Notably, MALT1-mediated degradation of Roquin-1 and regnase-1 appears to regulate Th17 differentiation in a manner dependent on TCR signal strength (102, 103). Roquin-1 naturally represses key proteins involved in CD4 Th17 differentiation (such as IκB_NS_, ICOS, IRF4, and Regnase-1), but its cleavage by MALT1 is necessary for derepression of IκB_NS_, which acts on nuclear NF-κB complexes. This cleavage occurs only under strong TCR signaling conditions (102) and similarly applies to Regnase-1 (103). Consistent with these findings, Roquin-deficient T cells are impaired in Th17 differentiation (103), as are mice deficient in the atypical IκB protein IκB_NS_ (104).

As previously mentioned, PKCθ is the most proximal TCR NF-κB signaling intermediate, phosphorylating CARMA1 to facilitate the formation of the CBM complex. Yet, in stark contrast to other downstream NF-κB signaling intermediates, PKCθ deficiency does not affect Th1 differentiation but is linked to defects in Th2 differentiation (105, 106). Some reports also indicate a connection between PKCθ and Th17 differentiation, potentially via STAT3 or by overriding Foxp3 suppression through SRC1 phosphorylation (107, 108). Interestingly, PKCθ-deficient T cells show specific impairment in TCR-dependent NF-κB signaling while retaining functional TNF and IL-1-driven NF-κB signaling (109). This suggests that PKCθ impacts Th differentiation at priming (when antigen is present) either independently of NF-κB or by altering the TCR signaling thresholds required for differentiating into specific Th subsets.

Furthermore, recent studies have explored the role of non-canonical NF-κB signaling in Th differentiation and illuminated the potential roles of upstream NF-κB signaling intermediates that are independent of the NF-κB transcription factors. For example, studies using *Ikka* ^AA^ knock-in mice deficient in IKKα activation found that nuclear IKKα selectively associates with the *Il17a* locus, promoting histone H3 phosphorylation and transcriptional activation in a NF-κB –independent manner (110). Additionally, mice deficient in NIK or Rag deficient mice reconstituted with NIK deficient CD4 T cells show impaired Th17 differentiation (but not differentiation to other Th subsets) and are resistant to EAE (111). This is in part due to their regulation of RORγt and IL-23R via the cooperation of IL-6R and TCR signals (111). A recent report also indicated that inhibition of IAPs (which leads to NIK stabilization and subsequent induction of p52-RelB) impairs Th17 differentiation and favors Th2 differentiation instead (112). These findings are consistent with a previous study reporting that p100-deficient T cells fail to differentiate into Th17 cells (113). Thus, both canonical and non-canonical NF-κB signaling can regulate Th differentiation. Although it is unclear whether this is related to a crosstalk between both pathways, inhibition of IAPs appear to shift canonical toward non-canonical signaling in CD4 T cell differentiation. This suggests that while non canonical NF-κB signaling is important for the initiation of Th cell differentiation for certain subsets (such as Th17), a shift toward non-canonical signaling may divert CD4 T cells away from Th17 differentiation and inflammatory IL17 or GM-CSF (114) production and toward the generation of more regulatory Th2 and Treg subsets (112).

Regarding Tfh differentiation, there are currently no established links between T cell-intrinsic non-canonical NF-κB signaling intermediates and Tfh development. It is important to note that many studies discussed above did not utilize inducible systems to control non-canonical signaling temporally or to restrict its activation exclusively to CD4 T cells. Given that non-canonical NF-κB signaling regulates lymphoid organogenesis, these studies could not rigorously exclude other non-CD4 T cell-intrinsic defects. This complicates the interpretation of the results. Additionally, *in vitro* and *in vivo* findings due to altering non canonical NF-κB signaling in T cells do not often align. This highlights the potential relevance of TNFR members (**Figure 2**) that are not included in *in vitro* studies.

In summary, future studies should adopt more rigorous approaches to clarify the T cell-intrinsic interplay of canonical and non-canonical NF-κB signaling and its relationship with TNFR costimulatory receptors in the context of disease.

### CD8 T cell differentiation

PKCθ is required for CD8 effector responses *in vivo*, particularly in the context of weak innate signals (115) (**Figure 2**). By contrast, CARMA1 deficiency has a more profound impact on CD4 T cells than on CD8 T cells. Supporting this notion, TCR mutant T cells that are impaired in CBM complex formation can still undergo normal CD8 effector differentiation during *Listeria monocytogenes* infection (64). However, in the context of cancer, one study has indicated that CARMA1 is critical for controlling transplantable tumors (116); although, it remains unclear whether this effect is solely due to the deficiency of CARMA1 in CD8 T cell function or is also influenced by the lack of CARMA1-deficient CD4 T cell help to CD8 T cells, as suggested by a recent investigation (69). Given the limited studies using CD8-restricted conditional or inducible BCL10, MALT1, or any of the NF-κB subunit mouse models, the specific roles and temporal regulation of CD8 effector differentiation remain unresolved in the field (**Figure 3D**).

### γδ T cell differentiation

γδ T cells can differentiate into Th1- or Th17-like γδ T effector cells, with γδ T17 cells playing a crucial role in antibacterial immunity (117–119). Unlike induced NKT cells, NF-κB signaling is dispensable for the generation of γδ T cells; however, it is necessary for IL-17 production by γδ T17 cells in response to LTβR engagement. RelA regulates the expression of LT ligands in accessory thymocytes, while RelB, acting downstream of LTβR, is required for the expression of the transcription factors RORγt and RORα, facilitating the differentiation of thymic precursors into γδ T17 cells (120).

## NF-κB signaling, thymic and peripheral Tregs

A substantial body of literature has established the critical role of the NF-κB signaling cascade in the generation and function of regulatory T cells (Tregs). Tregs can be categorized into two types: thymic Tregs (tTregs), which arise during thymic selection (43, 121), and peripheral Tregs (pTregs), generated from conventional CD4 T cells that attain stable Foxp3 expression in tissues such as the gut mucosa. Additionally, a third type known as *in vitro*-induced Tregs (iTregs) can be generated by differentiating conventional CD4 T cells under specific conditions. Regulatory CD4 T cells are phenotypically characterized by high expression of the IL-2 receptor alpha chain (CD25) and the master transcription factor Foxp3. Foxp3 directly and indirectly regulates a multitude of suppressive mechanisms that control inflammation and autoreactivity. Among these mechanisms are the expression of IL-10, Granzyme B, and CTLA-4, which hampers dendritic cell (DC) activation. Expression of CD25, instead, acts as a sink for IL-2, limiting the expansion and activation of CD8 T cells, natural killer (NK) cells, and innate lymphoid cells (ILCs). Remarkably, Tregs have also recently been recognized for their roles in tissue repair and wound healing, employing mechanisms that are distinct from immunosuppression [recently reviewed in (122, 123)].

The specific role of NF-κB in the generation of thymic Tregs is well established. It predominantly relies on TCR-mediated NF-κB signaling. Tregs that develop in the thymus are selected based on the strength and affinity of their TCR interactions with self-peptide-MHC complexes. Moderately strong interactions guide immature CD4 T cells toward becoming tTregs, endowing them with suppressive competence (43, 121). Interestingly, the selection processes for tTregs and naive T cells do not equally dependent on NF-κB signaling. Deficiencies in various intermediates of TCR-dependent NF-κB signaling (such as PKCθ, CARMA1, BCL10, TAK1, and IKKβ) significantly reduce the number of tTregs without compromising the generation of conventional CD4 or CD8 T cells. The observation that thymic Treg development is restored in TAK1 and Carma-1 deficient mice crossed with transgenic mice expressing a constitutively active form of IKKβ (IKKEE-Tg) further reinforces the idea that NF-κB signaling is sufficient to support Treg development (48, 124–128). However, it is less clear whether other stimuli (aside from antigens) driving NF-κB signaling also support Treg development in the thymus. A study by Mahmud et al. demonstrated that inhibition of TNFRSF members GITR, OX40 and TNFR2 signaling abrogates tTreg development in a TAK1-dependent manner (129). This study also indicated that TCR signaling regulates the levels of these TNFRSF members, allowing thymocytes to respond more efficiently to GITRL, OX40L and TNF signal and thereby facilitating their differentiation into tTregs (129). Importantly, this study did not clarify whether the role of these TNFRSF members in tTreg development is dependent on NF-κB signaling (129). Therefore, it is possible that TAK1 regulates tTreg development in an NF-κB -independent manner, similar to the mechanisms observed in SP thymocytes (50).

Recent reviews underscore the critical roles of both c-Rel and RelA in the development of Tregs within the thymus and their stability in the periphery. RelA deficiency leads to a complete blockade in the development of RORγt+ Treg cells (130, 131). However, studies involving conditional deletion of c-Rel or RelA specifically within developing and mature Tregs have revealed distinct roles in Treg biology. These studies demonstrate that c-Rel is critical for thymic Treg development and the expression of GITR and CD25. In turn, p65/RelA is essential for the differentiation of tTregs and also for peripheral Treg differentiation and maintenance of immune tolerance (131). Mechanistically, the NF-κB subunit c-Rel binds to the TCR-responsive conserved non-coding enhancer sequence 3 (CNS3) in precursor Tregs, significantly contributing to the expression of Foxp3 (128, 132)(**Figure 3A**). Additional studies indicate that p65/RelA enhances Foxp3 functions through the kinases Stk3 and Stk4. The group of Hongbo Chi found that both kinases are important for Foxp3-mediated peripheral tolerance by regulating the levels and function of peripheral (but not thymic) Tregs (133). These kinases modulate IL-2/STAT5 signaling, which is crucial for CD25 and Foxp3 expression, Treg lineage survival, stability, and function (133). Furthermore, Chatila’s group showed that Stk3 and Stk4 are integral components of a TCR/NF-κB signaling network, wherein TCR signaling leads to the nuclear translocation of Stk4. Stk4 forms a complex with p65/RelA and Foxp3, facilitating the control of the Treg transcriptional program through the phosphorylation of Foxp3 at Ser418 (134). Thus, the canonical NF-κB pathway emerges as a master regulator of Treg development and function.

The non-canonical NF-κB pathway has also been linked to Tregs, albeit in an unexpected manner. Conditional deletion of NFKB2 and/or RelB (in both total T cells and Tregs) has revealed a critical role for NFKB2 in maintaining Treg homeostasis (135). Specifically, NFKB2 limits the formation of RelB complexes that regulate cell-autonomous Treg expansion in the periphery. This uncontrolled Treg expansion is restored upon deletion of RelB (135). In contrast to this, other studies have shown that forced activation of the alternative pathway (through deletion of TRAF3 (Traf3−/− mice) or overexpression of NIK (NIK-transgenic [Tg] mice)) results in an increase in Treg numbers (136, 137). These findings suggest that the primary function of NFKB2 is to prevent aberrant RelB activation, with little or no role in regulation of Tregs under steady-state conditions. Given that NIK and IKKα can also activate the canonical pathway (**Figure 2**), it is possible that the effects of upstream intermediates in alternative NF-κB signaling on Tregs primarily operate through the canonical pathway (involving p65/c-Rel rather than through the non-canonical NF-κB subunits). Furthermore, the specific roles of the alternative pathway in high-inflammatory contexts, such as chronic infections where TNF receptor superfamily members (e.g., OX40, GITR) are actively engaged, still require further elucidation (138, 139). For instance, unprocessed NFKB2 (p100) may be induced by acute TCR signaling and could function as an inhibitor of RelB, thereby limiting Treg expansion until inflammatory, TNF receptor-mediated signaling activates the processing of p100 into p52. This processing may enable the formation of RelB-containing complexes that promote Treg expansion, potentially affecting their functionality (135, 139).

NF-κB signaling is also essential for the suppressive function of Tregs. For example, conditional deficiency of PDK1 in CD4 T cells results in reduced Treg levels and impaired function (**Figure 2**). This leads to uncontrolled proliferation of TCRγδ cells and expression of IL-17, ultimately triggering colitis (140). It remains uncertain, however, whether PDK1’s regulation of Treg function depends exclusively on NF-κB signaling or involves other pathways, such as mTOR. Interestingly, the downstream target of PDK1, PKCθ, has been found to inhibit Treg function (141, 142). Research indicates that PKCθ (**Figure 2**) localizes differently within Tregs compared to conventional CD4 T cells at the immunological synapse, resulting in a bias toward Treg programming over alternative Th effector fates (141, 142). Consistent with this observation, inhibition of PKCθ enhances the suppressive capacity of Tregs, restores the impaired function of Tregs from rheumatoid arthritis patients, and blocks autoimmune responses in mouse models of colitis (143).

Furthermore, the Ubc13/IKK axis plays a positive role in Treg suppressive function as conditional ablation of Ubc13 in Foxp3+ Tregs leads to multi-organ inflammation due to uncontrolled activation of conventional T cells (**Figure 2**) (144). Additionally, atypical members of the IκB family, such as Bcl3, IκBζ, and IκB_NS_, also contribute to Treg biology by fine-tuning the transcriptional activity of NF-κB in the nucleus. Unlike classical IkBs, these atypical members are not degraded but are induced upon stimulation (29). They regulate dimer exchange, recruit histone-modifying enzymes, and stabilize DNA-bound NF-κB dimers (29). Specifically, deletion of Bcl3 in Tregs abrogates its interaction with p50, promotes the formation of RORγt+ Tregs, and enhances mice’s resistance to induced colitis (145–147). By contrast, deletion of IκBζ in Tregs results in impaired Treg function, potentially due to the inhibition of Foxp3 promoter activation (97, 148). Taking altogether, while the NF-κB signaling pathway is crucial for the generation and function of Tregs, not all pathway members share identical roles. This suggests that complex, non-redundant mechanisms are involved in fine-tuning this important T cell subset.

## NF-κB signaling, T cell memory generation and maintenance

Memory T cells, long-lived plasma cells and memory B cells, are essential components of protective immunity. These memory lymphocytes have a unique ability to survive for extended periods within the body and, respond rapidly upon re-exposure to the same antigen that prompted their generation. This antigen can originate from various sources, including microbes (infections), self-tissues (autoimmunity or cancer), or foreign tissues (transplants). Consequently, memory T cells serve not only as powerful tools for the immune system to prevent disease but also as mediators of autoimmune pathology and transplant rejection. Memory T cells can recognize highly conserved antigens that are not accessible to antibodies. Interestingly, these antigens are often less prone to mutation when compared to epitopes recognized by antibodies or B cell receptors (BCRs). This allows memory T cells to effectively circumvent the immune escape of rapidly mutating pathogens (such as influenza or SARS-CoV-2 viruses). In the context of cancer, the generation of tumor antigen-specific memory CD8 T cells also holds significant promise for preventing metastasis and recurrence. Therefore, eliciting memory T cells is a desirable goal in vaccine development and cancer immune therapies. NF-κB signaling plays a crucial role in the development and maintenance of T cell memory. However, the specific mechanisms and NF-κB-driven signals that regulate these processes during immune responses are still not well understood.

The pool of memory T cells in an individual includes various subsets, such as central memory T cells (T_CM_), effector memory T cells (T_EM_), resident memory T cells (T_RM_), lymphoid tissue-inducible (LIP) memory cells, and virtual memory T cells (149). These subsets can be broadly classified based on their locations. Circulating memory T cells reside in secondary lymphoid organs (T_CM_) or move in and out of tissues via the bloodstream (T_EM_). In contrast, T_RM_ cells stay localized within specific tissues and provide immediate protection against infections (150–153) while limiting viral transmission (154). The presence of T_RM_ cells in tumors and draining lymph nodes is often associated with better cancer outcomes (155–157). Although circulating memory T cells are important for protective immunity and play essential roles in certain diseases (158), understanding the generation, function, and maintenance of both T_RM_ and circulating memory T cells is crucial. Elucidating these processes will help develop strategies to enhance immune responses and improve therapeutic outcomes across various diseases.

Early studies using genetic models, alongside analyses of human patients with mutations suggested that NF-κB signaling played a role in establishing long-term protective T cell memory pools (159, 160), However, the lack of inducible T cell-restricted NF-κB models has made it difficult to determine whether the observed defects in memory T cell levels result from early issues with T cell priming or proliferation, or from later problems in differentiation and survival. Likewise, these studies did not identify the environmental cues needed for T cell memory or whether NF-κB signaling is more important for specific memory subsets. In one of our studies, we discovered that T cells with TCRs that poorly activate NF-κB signaling—due to defects in forming the CBM complex—are unable to differentiate into circulating memory CD8 T cells during a systemic bacterial infection, despite normal effector differentiation (64, 161). Further research revealed that NF-κB signaling plays an unexpected role during the late phase of the immune response, particularly during contraction as memory precursors develop into memory cells (162). We used various genetic tools to show that p65/RelA is crucial for generating central memory CD8 T cells (T_CM_) during this phase (64). NF-κB signaling works in a feed-forward loop with the transcription factor Eomes and the kinase Pim1 (162). Deleting TCR-dependent NF-κB signaling or overexpressing an inactive form of PKCθ led to reduced Pim1 expression, loss of Eomes, and lower T_CM_ levels (162). Additionally, the loss of Pim1 activity resulted in decreased phosphorylated p65 NF-κB levels (162). Collectively, these findings indicate that during the early immune response, TCR signaling through NF-κB induces Pim1 expression. Once the antigen is cleared, Pim1 works with Eomes and NF-κB to sustain the necessary levels of NF-κB required for generation and maintenance of circulating T_CM_ cells.

The role of non-canonical NF-κB signaling in T cell memory has been explored in two studies (163, 164). Rowe et al. utilized NIK-deficient CD4 T cells in the context of LCMV infection and were the first to demonstrate that NIK deficiency impairs both the accumulation of effector and memory CD4 T cells, although it does not affect their function (164). Notably, the memory defect was more severe than the effector defect. Since no difference in the generation of memory precursors was observed, it is possible that NIK play a role in the maturation or survival of CD4 memory cells. Similarly, Li et al. showed that NIK also regulates CD8 T cell memory (163). However, neither study addressed whether the observed memory defects were due to alterations in Treg development, lack of CD4 T cell help, impaired T cell expansion, or differential effects on circulating and resident memory T cell populations. Additionally, these studies did not assess whether the memory defects were NF-κB dependent or identify any TNFRSF members responsible for NIK activation. CD27, 4-1BB, and GITR are TNFR family members known to play important roles in T cell memory. 4-1BB and GITR signal through the canonical NF-κB pathway but they regulate tissue resident memory in an mTOR-dependent manner (165, 166). In contrast, CD27 can activate both the canonical and non-canonical NF-κB pathways (42, 167) and regulates Pim1 expression (168), which is critical for CD8 T cell memory survival via the TCR (162, 168) Thus, it is possible that TCR canonical signaling and CD27 non-canonical signaling converge at the level of Pim1 to regulate CD8 T cell memory.

Recently, we investigated how canonical NF-κB signaling contributes to the development and maintenance of different memory T cell subsets during influenza infection (169). We used two tetON IKKβ inducible mouse models that allow for the expression of both active and inactive forms of the IKKβ kinase (169). This approach enabled us to examine how NF-κB signaling influences CD8 T cells at various stages of the immune response to influenza. Our findings revealed distinct roles for IKKβ/NF-κB signaling in generating influenza-specific circulating memory T cells and resident memory T cells (T_RM_). Higher levels of IKKβ/NF-κB signaling after day 8 post-infection were associated with an increase in circulating memory CD8 T cells. However, this was accompanied by a decrease in T_RM_ in the lung, which negatively impacted protective immunity against influenza. In contrast, inhibiting IKKβ/NF-κB signaling led to a reduction in circulating memory T cells while increasing T_RM_ populations in the lung (169). We found that NF-κB signaling limits the transcriptional program of T_RM_ cells without affecting their recruitment to the lung, likely due to inhibiting local TGF-β signaling (169). Additionally, TNF acts as a driver of NF-κB signaling during influenza infection and negatively influences T_RM_ cell generation. Blocking TNF increased the number of influenza-specific CD8 T_RM_ cells in the lung and improved TGF-β signaling along with the expression of Runx3 (169). It remains unclear whether high or chronic levels of TNF affect CD8 T_RM_ generation in other contexts, as TNF levels often rise in certain infections and chronic inflammatory diseases. Other cytokines and TNF receptor superfamily members may also modulate NF-κB levels, influencing the size of circulating and resident memory CD8 T cell pools.

While previous studies employing single-cell RNA sequencing have indirectly suggested the importance of NF-κB for tissue-resident memory (151, 170–172), our research demonstrates that NF-κB signaling is crucial for memory diversity and T cell-mediated immunity (169), (**Figure 3E**). However, the exact mechanisms by which the transcription factor NF-κB differentially regulates circulating and T_RM_ cells remain to be determined, especially regarding the involvement of NF-κB subunits.

Establishing an efficient CD8 T cell memory pool depends on two key factors: effective differentiation and persistent survival. We have discovered that NF-κB signaling also plays a crucial role in maintaining CD8 T cell memory and positively influences both circulating and tissue-resident memory (T_RM_) cells. When NF-κB signaling is inhibited in CD8 memory T cells, both influenza-specific circulating and lung-resident memory T cells diminish. This loss is likely due to decreased levels of survival factors like Bcl-2 and IL-15R (CD122) (169). On the other hand, increasing IKKβ/NF-κB signaling in memory T cells increases the levels of Bcl-2 and IL-15R, which enhances both T_RM_ and central memory (T_CM_) populations (169). These findings, altogether, indicate that canonical NF-κB signaling regulates the generation and maintenance of memory CD8 T cells in distinct ways; although, the specific mechanisms still need further investigation.

In summary, NF-κB signaling is critical for forming and maintaining memory T cells. Its role is complex and varies depending on the level of NF-κB activation and the stage of T cell differentiation. The studies we discussed provide insights into how tuning NF-κB signaling can aid to independently regulate the levels of circulating and resident memory CD8 T cells. This approach could be particularly useful in certain diseases, allowing to selectively deplete T_RM_ while preserving circulating memory cells. Such a strategy may help reduce pathogenicity without sacrificing protective immunity.

## NF-κB signaling and T cell exhaustion or dysfunction

T cell exhaustion or dysfunction refers to effector T cells that are persistently stimulated by antigens and costimulatory signals, typically in chronic infections, cancer, or autoimmunity (173). Exhausted T cells gradually lose their effector functions, such as cytokine secretion and the ability to kill target cells. This decline is caused by an accumulation of inhibitory signals, marked by high levels of inhibitory costimulatory molecules like PD-1, CTLA-4, Tim-3, LAG-3, TIGIT, and 2B4 (173). Immune checkpoint blockade (ICB) is a therapeutic strategy that uses antibodies to inhibit PD-1 and CTLA-4 signaling. This approach reduces inhibitory signals and helps rejuvenate exhausted T cells, restoring their effector functions (174). However, ICB is not equally effective for all patients. It works best in T cells that have not reached terminal exhaustion, particularly in stem-like or Ly108+ progenitor exhausted T cells located in draining lymph nodes, and CX3CR1 effectors migrating to tumors (175). Recent studies that employed single-cell RNA sequencing have shown that the NF-κB family (including REL, RELB, NFKB1, and NFKB2) is more active in Ly108+ T progenitors of exhausted T cells (176, 177). This suggests that NF-κB signaling is crucial for the development and maintenance of these cells (177). Additionally, different SWI/SNF complexes epigenetically regulate the various stages of T cell exhaustion. The BAF complex promotes the differentiation of exhausted T cell progenitors into an effector-like subset, while the PBAF complex prevents terminal exhaustion, helping to maintain T cell progenitors. Although the exact mechanisms behind the balance between BAF and PBAF are not completely clear, it is likely that NF-κB signaling connects these processes with T progenitors (178, 179). Further research is essential to explore these possibilities and elucidate the complex dynamics of NF-κB signaling in T cell exhaustion.

The potential for NF-κB signaling to regulate T cell exhaustion is significant, but no study has definitively established its role. It’s still unclear which specific components of the NF-κB signaling pathway are critical and how they might function during the differentiation of exhausted T cells. Recent research suggests that 4-1BB-dependent NF-κB signaling can help T progenitors differentiate into effector-like cells and promote their proliferation through RelA and c-Rel signaling. Notably, using 4-1BB therapy alongside anti-PD-1 treatment (but not before treatment or in established tumors) led to reduced tumor burden and longer survival (180). If future studies confirm that NF-κB signaling is essential for maintaining tumor-specific T cells at low exhausted stages (i.e. progenitors of exhausted T cells) this could open new avenues for treatment strategies.

## NF-κB signaling, transcriptional, epigenetic and metabolic regulation

NF-κB signaling plays a crucial role in T cell responses at the transcriptional, epigenetic, and metabolic levels (15, 181–183). T cell differentiation involves changes in chromatin structure, which affect gene accessibility. Each transition between naive, effector, and memory T cells includes chromatin remodeling, shifting from compacted (silent) to active states (184–186). This remodeling is controlled by chemical modifications to histones and DNA. Various enzymes act as “writers” (which add acetyl or methyl groups), “erasers” (which remove them), and “readers” (which bind to modified chromatin). Chromatin modifications generally precede gene transcription and are influenced by epigenetic regulators (9, 187–190). Typically, histone acetylation promotes transcription, while methylation is linked to gene silencing.

NF-κB family members are key players in chromatin remodeling. They can recruit and position chromatin modifiers to specific genes (191, 192). For example, GITR-dependent NF-κB signaling helps induce Tregs to become Th9 cells by allowing p50 to recruit deacetylases HDAC1 and Sirt1 to the Foxp3 locus. This process leads to the closure of the Foxp3 locus, enabling induced Tregs to differentiate into inflammatory Th9 cells (193). Research shows that RelB also aids in regulating chromatin in activated T cells. In Th17 conditions, OX40 stimulation represses IL-17 expression by directing RelB to the IL-17 locus, leading to the trimethylation of H3K9 and the closure of the IL-17 locus (192). Conversely, RelB can promote Th19 differentiation by recruiting acetyltransferase p300/CBP to the IL-10 locus (194).

Mounting evidence also suggest that the specific partners of NF-κB subunits can separate their epigenetic and transcriptional tasks (195). However, the mechanisms behind these separations and the factors determining the interaction with specific chromatin modifiers are still unclear. New techniques like ATAC-seq, CUT&RUN, and CUT&Tag offer great promise for advancing our understanding in this area. Recent research using single-cell ATAC-seq has shown that the collaboration of RelA or c-Rel with IRF-3 and MAPK factors during TLR4 responses enhances remodeling selectivity at specific genomic regions (196).

**Figure 4:**
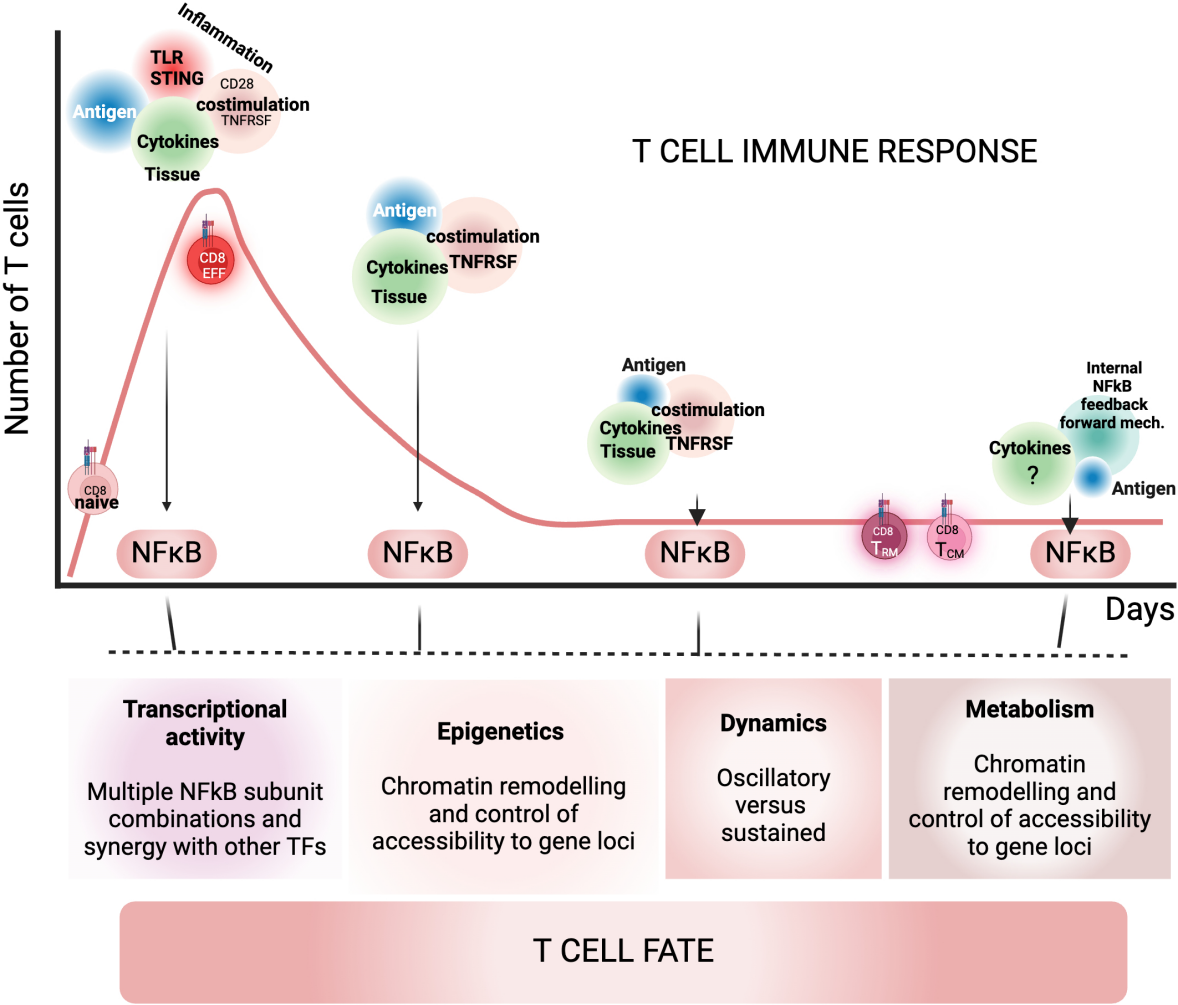
NF-κB signaling triggers and NF-κB mechanisms that regulate CD8 T cell fate during a T cell immune response. This cartoon illustrates the key triggers and mechanisms of NF-κB signaling that influence CD8 T cell fate during a T cell immune response. The primary triggers of NF-κB signaling include antigen recognition, inflammation (including costimulation and pro-inflammatory cytokines), and local cytokines. As levels of antigen and inflammation decrease, other factors—such as tissue cytokines, TNF receptors, and internal NF-κB forward loops established early in the response—assume a more prominent role in determining T cell memory fate during the late phase of the immune response. The functions of NF-κB that operate in T cells are depicted in the cartoon; however, the specific mechanisms by which these factors regulate T cell fate, either independently or synergistically, remain largely unknown. This figure was created using BioRender.com.

T cell metabolism is essential for T cell differentiation, and NF-κB signaling plays a significant role in this process (197, 198). For example, NIK-deficient tumor-infiltrating CD8 T cells do not shift toward glycolysis, which leads to a loss of effector function and impaired tumor control. Notably, NIK operates independently of NF-κB subunits by regulating levels of intracellular reactive oxygen species (ROS) (199). While there is limited information on how other NF-κB signaling intermediates affect T cell metabolism, some studies suggest a link between NF-κB signaling and mTOR in regulating metabolic changes critical for T cell differentiation (197, 198). mTORC1, which senses nutritional cues, is essential for naive CD8 T cells to transition to glycolysis, ensuring they have enough energy for effector functions. Research by Kane’s group has found that CARMA-1 and MALT1, in addition to activating IKKβ, also contribute to mTORC1 activation, which is vital for CD4 T cell proliferation (200). Moreover, Pearce et al. demonstrated that T cells lacking TRAF6—a ligase important for IKKβ activation in response to antigen, TNF, and IL-1R stimulation—fail to differentiate into memory T cells due to impairments in fatty acid metabolism after IL-2 withdrawal during maturation. They found that treatment with metformin and rapamycin could help recover this memory defect (201). However, it remains unclear whether TRAF6 functions independently of NF-κB signaling in this context. It’s also worth noting that mTOR signaling can influence NF-κB activity (202). Given the significant role of NF-κB signaling in regulating metabolism in other cell types, it is highly likely that NF-κB also plays a crucial role in the metabolic shifts necessary in T cell function and differentiation. Further investigation to fully elucidate the underlying mechanisms (**Figure 4**).

## Dynamics of NF-κB signaling and their impact in T cell fate decisions

How does the NF-κB signaling network inform a T cell’s decisions, such as committing to a specific fate, secreting certain cytokines over others, or surviving for extended periods? This is a complex question with many unanswered aspects. T cells may encounter a variety of NF-κB signaling triggers some of which may converge on the same canonical or non-canonical pathway. Both the level and timing of these signals, along with other transcriptional and epigenetic factors, are important. Additionally, T cells at different developmental stages may respond differently to the same stimuli (**Figure 4**).

A foundational study by Hoffman and Baltimore demonstrated that NF-κB signaling operates not as a simple ON/OFF mechanism but rather exhibits a “tidal” pattern (203, 204). This means that NF-κB signaling creates peaks and valleys of activation in an oscillatory manner which the cell interprets for decision-making (203). The IκB family serves as the primary regulator of NF-κB activation and is also induced by NF-κB signaling. Newly synthesized IκBα binds to p50 and p65 in the nucleus, inhibiting their transcriptional activity and shuttling the IκBα-p50-p65 complexes into the cytosol. This process keeps NF-κB inactive until the next signaling burst occurs (205). Other IκB family members also play a role in modulating the dynamics of NF-κB activity (205).The dynamics of NF-κB can vary depending on the ligand, dosage, and duration of cell stimulation, leading to different gene expression profiles and epigenetic changes related to T cell outcomes. This has not been fully demonstrated in T cells. However, studies in other cell types have shown that prolonged TNF stimulation can lead to oscillatory NF-κB responses (203, 204), while prolonged LPS stimulation results in more sustained NF-κB activation with fewer oscillations (206, 207). These differences in NF-κB dynamics impact various epigenetic programs. For instance, sustained non-oscillatory responses in macrophages promote chromatin remodeling and activate latent enhancers that regulate immune response genes. Conversely, oscillatory responses are associated with maturation-related genes in B cells and chemokine production in fibroblasts (208).

Additionally, recent studies suggest that chronic TNF-derived NF-κB signals in the lung inhibit the generation of tissue-resident memory T cells, while sustained signals in the lymph nodes increase the levels of circulating memory T cells (169). This raises the question of whether these effects are linked to different NF-κB dynamics influenced by various triggers.

Growing evidence suggests that the dynamics of NF-κB signaling—whether oscillatory or sustained—play a crucial role in determining the accessibility of NF-κB-dependent genes and influencing cell fate decisions (43, 209). However, the upstream mechanisms that govern these dynamics are not well understood. One key factor is the balance between IKK complex activation and inhibitory mechanisms. Molecules involved in the formation, ubiquitination, and activation of the IKK complex are essential for this balance, along with the activity of inhibitory proteins such as A20. For instance, variations in stimulus dosage, such as TNF, can affect NF-κB signaling dynamics through negative feedback loops involving A20 and IκBα (13). A20, like IκB, is induced by NF-κB signaling and helps degrade upstream intermediates, including RIP1, TRAF6, and MALT1. This degradation inhibits further activation of the IKK complex. Additionally, other negative regulators of the NF-κB pathway, such as CYLD and OTULIN deubiquitinases, may also contribute to the regulation of NF-κB signaling (210, 211) (**Figures 1C, D**).

Additional mechanisms for controlling NF-κB dynamics include preventing spontaneous activation by regulating the processing of p100 or p105 (212, 213). Temporal separation of activation signals and their receptors can occur in endosomes through the ESCRT pathway (213). Moreover, signaling can be modulated by microRNAs and long non-coding RNAs (214, 215).

Thus, the field of NF-κB biology is approaching a pivotal moment. New inducible NF-κB animal models, along with advances in single-cell RNA sequencing (scRNA-seq) and single-cell ATAC sequencing (scATAC-seq), will significantly enhance our understanding. These technologies, combined with computational and signaling network models, will help elucidate how NF-κB signaling in T cells influences decisions that can either protect against disease or contribute to its progression.

## NF-κB, cell type and temporal targeting and therapeutics

NF-κB signaling is vital for T cell biology, especially in the development of T cell subsets that, when deregulated, contribute to various diseases. As discussed throughout this review controlling NF-κB signaling intermediates in a temporal and specific manner presents therapeutic opportunities for treating infections, cancer, autoimmunity, and transplantation. Selectively targeting specific NF-κB subunits could lead to effective treatments that provide benefits without compromising other NF-κB functions. For example, targeting c-Rel may enhance antitumor responses because it is crucial for creating and maintaining activated Tregs that accumulate at tumor sites and impair the function of anti-tumor T cells (216).

However, precise targeting of specific T cell types is essential for effectiveness. Early deletion of c-Rel in conventional CD4 T cells can increase tumor burden and reduce responses to checkpoint blockade (69). In studies, deleting c-Rel during the first five days after tumor implantation reduced CD4 T cell priming, resulting in more naive T cells but impaired effector functions (69). While in this study the later roles of c-Rel and RelA in the antitumor response not explored, it is plausible that inhibiting c-Rel at later stages may not impact conventional T cells but rather affect activated Tregs, potentially enhancing antitumor responses. In related research, the Mempel group found that disrupting the CBM complex—through the genetic deletion of CARMA1 or inhibition of MALT1—promoted Treg activation toward a Th1/IFN-γ producing phenotype. This change, combined with PD-1 blockade, led to the rejection of PD-1 resistant tumors (217). Conversely, other studies suggest that IKKβ or CARMA1 activation can limit tumor growth in a CD8-dependent manner (116, 218, 219). Thus, when evaluating NF-κB inhibitor therapeutics, it is vital to consider their effects on all components of the antitumor response and the timing of their actions. This approach could reveal new opportunities for using multitarget inhibitors effectively.

In autoimmune contexts, early inhibition of p65/RelA (but not other subunits) may be beneficial. Its absence in CD4 T cells has shown complete protection against autoimmunity in an experimental autoimmune encephalomyelitis (EAE) mouse model, which relies on Th17 (69). There is less information on effectively targeting NF-κB signaling to reduce transplant rejection, as current alternatives to broad immunosuppressive treatments often affect multiple immune cells. The potential for selectively activating or deactivating NF-κB subunits to manage diseases with deregulated T cell responses—while preserving T cell memory—also deserves further exploration.

Numerous NF-κB inhibitors are being developed and tested in clinical trials for various treatments. Additionally, existing drugs that affect NF-κB activity are being repurposed, broadening options for targeting this pathway. This topic has been thoroughly reviewed recently, and we will not discuss it further here (55, 220). Nonetheless, it is important to mention that none of the options have achieved considerable success due to limited effectiveness or associated toxicity. As the field continues to clarify the specific roles of NF-κB subunits in disease, it is important to avoid broader inhibitions, such as those affecting proteasome activity or deubiquitination. Optimizing the timing of NF-κB inhibitor administration will enhance T cell response modulation and should consider the specific tissues and T cell subsets involved in disease progression or resolution.

## Conclusion and future perspectives

After nearly 40 years of research, the study of NF-κB signaling has not only flourished but continues to yield unexpected discoveries. This pathway has proven crucial in cell biology, regulating essential processes such as cell division, apoptosis, senescence, migration and effector function. NF-κB is classically considered a driver of inflammation and organogenesis but, for T lymphocytes, this signaling pathway is also a ruler of their fate. With the advent of new transgenic inducible animal models, previously contentious issues regarding the role of NF-κB in thymic selection, homeostasis, and CD4 T cell differentiation have been revisited. Recent findings have unveiled surprising roles for NF-κB in antitumor T cell responses, experimental autoimmune encephalomyelitis (EAE), and the development of T cell memory during infections. However, a critical question remains: how does the NF-κB signaling network enable T cells to make specific differentiation choices?

This may depend on how NF-κB signaling influences T cell epigenetics and metabolism. These areas, alongside NF-κB dynamics, are largely unexplored and require further investigation (**Figure 4**). Gaining a better understanding of how environmental triggers of NF-κB work during the immune response, along with their mechanisms, is essential and could lead to new therapeutic strategies. In addition to this, distinguishing between NF-κB-dependent and independent roles of signaling intermediates in the NF-κB cascade may clarify current knowledge, help identify effective therapeutic targets and anticipate potential side effects in treatments.

While this review does not cover it, existing literature has discussed human mutations in NF-κB pathway components and their disease implications (3, 34, 159, 221–223). Identifying mutations in key NF-κB molecules is an area that offers valuable insights and can enhance the rigor and impact of research using animal models, ultimately contributing to the development of new treatments for human diseases.

In conclusion, although significant progress has been made in understanding NF-κB signaling in T cell biology, many exciting areas remain to be explored. Advances in these fields will deepen our insights into immune responses and pave the way for innovative therapeutic strategies to combat diseases such as cancer, autoimmunity, and transplant rejection.
